# Expertise and decision-making in American football

**DOI:** 10.3389/fpsyg.2015.00994

**Published:** 2015-07-13

**Authors:** Adam J. Woods, Alexander Kranjec, Matt Lehet, Anjan Chatterjee

**Affiliations:** ^1^Center for Cognitive Aging and Memory, Institute on Aging – Department of Aging and Geriatric Research, University of FloridaGainesville, FL, USA; ^2^Department of Neurology, Center for Cognitive Neuroscience, University of PennsylvaniaPhiladelphia, PA, USA; ^3^Department of Psychology, Duquesne UniversityPittsburgh, PA, USA; ^4^Center for the Neural Basis of Cognition, Carnegie Mellon UniversityPittsburgh, PA, USA

**Keywords:** expertise, sports decision-making, spatial biases, American football, pass interference

## Abstract

In American football, pass interference calls can be difficult to make, especially when the timing of contact between players is ambiguous. American football history contains many examples of controversial pass interference decisions, often with fans, players, and officials interpreting the same event differently. The current study sought to evaluate the influence of experience with concepts important for officiating decisions in American football on the probability (i.e., response criteria) of pass interference calls. We further investigated the extent to which such experience modulates perceptual biases that might influence the interpretation of such events. We hypothesized that observers with less experience with the American football concepts important for pass interference would make progressively more pass interference calls than more experienced observers, even when given an explicit description of the necessary criteria for a pass interference call. In a go/no-go experiment using photographs from American football games, three groups of participants with different levels of experience with American football (Football Naïve, Football Player, and Football Official) made pass interference calls for pictures depicting left-moving and right-moving events. More experience was associated with progressively and significantly fewer pass interference calls [*F*_(2,48)_ = 10.4, *p* < 0.001], with Football Naïve participants making the most pass interference calls, and Football Officials the least. In addition, our data replicated a prior finding of spatial biases for interpreting left-moving images more harshly than identical right-moving images, but only in Football Players. These data suggest that experience with the concepts important for making a decision may influence the rate of decision-making, and may also play a role in susceptibility to spatial biases.

## Introduction

### Decision-Making

On fourth down of Super Bowl XLV with less than a minute to decide the champions of the 2010 National Football League (NFL) season, the Pittsburg Steelers’ quarterback took the snap and passed the ball to his offensive wide receiver (Chase, 2011; Pereira, 2011; Rodgers, 2011)^1^. As the receiver leaped into the air to catch the ball, the Green Bay defensive cornerback stretched out his arm and knocked the ball away from the receiver’s grasping hands, however, the defender also made contact with the receiver during the defensive play. The Steelers fans in the stadium were vocal with demands that officials call pass interference on the Green Bay defender. A pass interference penalty would move the ball to the location of the foul and result in a first down – keeping the Steelers’ Super Bowl dream alive; however, the official nearest to the play made the decision that pass interference was not committed, a decision that is non-reviewable (Chase, 2011; Pereira, 2011; Rodgers, 2011). This pass would be the last offensive play of the game for the Pittsburgh Steelers in Super Bowl XLV. With 49 s remaining, Green Bay retook possession of the ball and allowed the game clock to expire (Pereira, 2011). Although analysis after the game would show that the call on the field was likely correct, the call itself played a major role in deciding the 2011 Super Bowl champions (Pereira, 2011). While fans, players, and officials all observed the same event, their interpretation varied widely. This is one of many such examples in the history of American football, and represents a common occurrence across decision-making in other sports and everyday life.

Our study investigated the influence of differing levels of experience with concepts important for pass interference on penalty decision-making criteria and spatial bias. The current study specifically evaluated the probability of making a pass interference (i.e., frequency) call and the presence of spatial (leftward vs. rightward) bias in penalty decisions on a task requiring participants to judge whether or not a pass-interference penalty was committed in a rapidly changing event depicted in a static image. We evaluated performance in persons with three levels of experience with pass interference in American football: (1) persons with little to no prior experience with American football, (2) college-level American football players, and (3) American football officials.

Decision-making requires the integration of perceptual and cognitive information (e.g., conceptual, contextual, etc.) to determine a course of action, whether in every day events (e.g., driving) or on the field of play (e.g., pass interference decisions; Kahneman, 2003; MacMahon and Starkes, 2008; Woods et al., 2012; Summerfield and de Lange, 2014). In many cases, decision-making requires rapid integration of this information (e.g., driving; Brooks et al., 2011; Hunt et al., 2011); however, perceptual information may be ambiguous due to the brevity of exposure to the event or other factors degrading the perceptual quality of the event (e.g., poor visual conditions; Brooks et al., 2011). Furthermore, prior experience and contextual information also play a significant role in forming a decision (MacMahon and Starkes, 2008; Woods et al., 2012). Research shows that response criteria for decision-making may remain stable or even improve as a result of prior experience and expertise (Ericsson, 2007; Lueddeke and Higham, 2011). Prior experience can also bias decision-making. For example, when people judge the causal nature of an event, their recent experience with causal and non-causal events shifts their concept of causal structure and results in a change in their response criteria when subsequently judging causality (Woods et al., 2012). While other research demonstrates the influence of experience on decision-making in other contexts (e.g., emotional, financial, causal decision-making; Carpenter and Yoon, 2011; Lueddeke and Higham, 2011; Woods et al., 2012), we sought understand the role of experience in decision-making in the context of sports-related events in American football. As decision-making is a central facet of human behavior, findings from the current study are relevant not only to pass interference penalty calls in American football, but may also be relevant for other sports-related decision-making and decision-making under similar conditions outside of sports (e.g., driving).

### Pass Interference

According to the NFL, there are at least six major, yet non-comprehensive, criteria for calling defensive pass interference (intentional contact by a defender that is not attempting to make a play on the ball, playing through the back of a receiver to make a play on the ball, impedance of a receiver’s arm movement to restrict his ability to catch the ball, extending an arm across the body of the receiver to restrict his catching ability, obstructing the receiver’s path without making a play on the ball, or turning the receiver’s body away from the ball before the pass arrives; NFL Digest of Rules, 2013). Officials make quick decisions on pass interference based on brief exposures to dynamic confrontations between players. Slow-motion television replays and post-game analyses do not always clarify the situation. This only serves to highlight the ambiguity involved in making pass interference decisions in football. As such, the ambiguity of pass interference in the NFL, as well as all levels of football, means that officials make decisions under difficult conditions and may be subject to errors. Since pass interference can result in one of the largest yardage penalties in American football, it is a penalty that can have a substantial impact on the outcome of a game. Furthermore, as pass interference calls cannot be reviewed, any factors that might influence officials’ decisions could have a large impact on the outcome of the game.

### Expertise

The 2012 NFL season is perhaps best known for the controversial use of replacement officials during an NFL-wide labor dispute, which caused officials to lockout. The tenure of replacement officials in the NFL was plagued with criticism for inaccurate penalty calls, poor clock management, inaccurate yardage penalization, and a bevy of additional errors (Holmes, 2012; Seifert, 2012; Staff, 2012; Wade, 2012). This is a prime example of the impact of expertise on sports decision-making. One of the most memorable examples occurred on September 24th 2012 during the final moments of a Monday Night Football game between the Seattle Seahawks and Green Bay Packers (Staff, 2012; Wade, 2012). In a last-second effort to win the game, the Seattle quarterback threw a long pass to his receiver in the corner of the end zone (2012 Packers-Seahawks officiating controversy, n.d.). As the ball came down, the Green Bay defender leaped into the air to intercept the pass (Holmes, 2012; Seifert, 2012; Staff, 2012; Wade, 2012). Following less than a second behind, the Seattle offensive receiver attempted to take the pass away from the Green Bay defender (Holmes, 2012; Seifert, 2012; Staff, 2012; Wade, 2012). The officials on the field judged that the Seattle receiver caught the ball and had possession of the ball at the end of the play (Holmes, 2012; Seifert, 2012; Staff, 2012; Wade, 2012). After a lengthy instant replay following the game ending play, the lead replacement official confirmed the call on the field (Holmes, 2012; Seifert, 2012; Staff, 2012; Wade, 2012); however, not only would television replay later demonstrate that the Green Bay defender caught the ball and had possession at the end of the play, but the Seattle receiver also committed offensive pass interference immediately before attempting to take the ball from the Green Bay defender (Holmes, 2012; Seifert, 2012; Staff, 2012; Wade, 2012). The prevailing argument was that inexperienced officiating cost the Green Bay Packers a victory (Holmes, 2012; Seifert, 2012; Staff, 2012; Wade, 2012). This example, among others, has been used to suggest that officials lacking the necessary experience and training to make calls at a given level of play may be more susceptible to error in sports decisions made in ambiguous conditions (Holmes, 2012; Seifert, 2012; Staff, 2012; Wade, 2012).

Prior research demonstrates the benefits of prior experience (i.e., training) in officials across a variety of sports (e.g., cricket, Sparrow et al., 2001; baseball, MacMahon and Starkes, 2008; soccer, Catteeuw et al., 2010; etc.). Research also demonstrates that expertise in one decision-making domain can transfer to decisions in similar domains (Rosalie and Müller, 2014; Loffing et al., 2015; Müller et al., 2015); however, when the characteristics of a decision-making domain vary significantly from the specific area of expertise, the likelihood of skill transfer decreases. While officials train to obtain expertise for penalty decision-making in American football, American football players do not possess this domain specific training. Thus, expertise in playing football may not necessarily transfer to expertise in football penalty decision-making; however, these same players possess more experience with the domain-specific information important for pass-interference calls as compared to persons with little-to-no prior exposure to American football.

The present study investigated whether different levels of experience with American football modulate decision-making processes for pass interference calls. We hypothesized (Hypothesis 1) that, like prior findings in other sports, expertise would influence participants’ response criteria for pass interference calls. Specifically, we hypothesized that increasing levels of experience with the concepts important for pass interference calls in American football would be associated with fewer overall pass interference calls. The current study also sought to understand the role of expertise in susceptibility to a potential source of bias: spatial direction.

### Spatial Biases in Sports Decision-Making

Recent research in soccer suggested that referees might be biased toward calling more fouls when plays move from left to right, rather than from right to left (Kranjec et al., 2010). In Kranjec et al. (2010), college soccer players were presented with pictures of player confrontations with a clear direction of motion (left–right or right–left). They were asked to decide if the picture depicted an unfair tackle. Soccer players were more likely to judge events with leftward motion as depicting fouls than the same stimuli presented with rightward motion. Kranjec et al. (2010) suggested that their results stem from a general bias for representing prototypical events from left-to-right and may possibly be related to repeated exposure to culturally specific reading habits (i.e., left to right reading and writing direction for English speakers). However, we lack a complete understanding of whether spatial biases are mainly the product of culturally –specific visual habits like reading direction (Tversky et al., 1991; Chatterjee, 2001; Maass and Russo, 2003; Dobel et al., 2007; Woods et al., 2013), body-specific motor habits related to handedness (Casasanto, 2011; Loffing et al., 2015) or the brain’s lateralization of spatial and attentional processing (Chatterjee et al., 1995; Chatterjee, 2011).

It remains unclear whether directional biases are generalizable to other sports events with inherent ambiguity. Furthermore, the role of experience in such biases remains unknown. Directional biases affecting decisions could have implications for officiating systems in any sport where (1) defensive and offensive players make contact and (2) officials have to make quick decisions under ambiguous circumstances. Modulation of bias through differing degrees of experience could provide insight into methods for mitigating perceptual biases in sports decision-making and other domains.

Similar to the logic of the prior soccer study, we hypothesized (Hypothesis 2) that English reading, football-knowledgeable participants would have lower thresholds for calling pass interference for leftward moving versus rightward moving events. This hypothesis was based on the idea that we conceptualize events as prototypically unfolding from left-to-right in space (Tversky et al., 1991; Chatterjee, 2001; Maass and Russo, 2003; Dobel et al., 2007; Kranjec et al., 2010). Right-to-left moving events should be perceived as atypical and relatively debased and viewers would be more likely to call a penalty (Kranjec et al., 2010). We further hypothesized (Hypothesis 3) that the influence of spatial biases will decline in people with greater levels of experience with American football (i.e., football officials). This hypothesis is supported by prior research demonstrating the benefits of expertise for sports-related decision-making (Sparrow et al., 2001; MacMahon et al., 2007; Loffing et al., 2015), as well as data demonstrating the benefit of transfer of expertise to a task with demands similar to the expertise domain (MacMahon et al., 2007; Rosalie and Müller, 2014; Loffing et al., 2015; Müller et al., 2015). To address our hypotheses, we assessed three groups of participants with different levels of experience with American football: (1) participants with little to no prior knowledge of American football, (2) American football players, and (3) American football officials.

## Materials and Methods

### Ethics Statement

This study was approved by the Institutional Review Board and the office of Athletics for Compliance at the University of Pennsylvania. The work was conducted according to the principles expressed in the Declaration of Helsinki. Written informed consent was obtained from all participants. Participants were paid for their participation.

### Participants

Three groups of 16 right-handed native English speaking male participants were recruited for this study (*N* = 48). Groups were selected based on their level of experience with American football. Sixteen participants were male college students with no prior experience playing American football, limited exposure attending, or watching televised football games, and no experience officiating (Football Naïve group; mean age = 20.1 years). Sixteen participants were male members of the University of Pennsylvania’s varsity football team with no experience officiating American football (Football Player group; mean age = 19.8 years). Sixteen participants were male National Collegiate Athletic Association (NCAA) football officials recruited at a Philadelphia-based annual officiating workshop (Football Official group; mean age = 54.5 years). Each participant completed a 12-item questionnaire created for the current study assessing their playing/watching experience, knowledge of NFL rules, teams, and players. This measure was intended to distinguish between people knowledgeable of American football and those with little to no prior knowledge. The questionnaire consisted of eight multiple-choice questions and four 5-point Likert scale response questions. Please see Supplemental Materials for the full questionnaire. The Football Official group were administered four additional short answer questions ascertaining their degree of experience officiating college-level American football games.

### Stimuli

All photos used for stimuli depicted scenes from high school, college, and professional football teams across the US. Photographs were obtained from Google Image and chosen according to four criteria: (1) scenes depicted only two athletes directly involved in the play; (2) the ball was in the air and not in contact with either player; (3) of the two players, one player was clearly the offensive player attempting to catch the ball, and the other was clearly the defensive player attempting to disrupt completion of the catch (i.e., one player was clearly closer to the ball than the other player and making a play for the ball); and (4) pictures depicted a strong implied rightward or leftward direction of movement. Twelve of the final 95 stimuli showed the ball already in possession of the offensive receiver (i.e., violating criteria 2) to provide clear instances where pass interference had not occurred and give participants a spectrum of events similar to what officials experience during the course of a game. Thus, all stimuli did not depict pass interference. Furthermore, stimuli where the ball was not in possession of a player (*n* = 83 stimuli) were specifically chosen because the picture depicted an ambiguous scenario difficult to determine as pass interference. Our stimuli were chosen to address questions regarding response criteria and spatial biases in sports decisions, not the accuracy of the calls themselves. Thus, these 83 stimuli were not chosen relative to accuracy of pass interference. Pictures were specifically chosen to depict visual perceptual information officials are exposed to on the field. Numbers and letters were removed from uniforms and backgrounds using Photoshop. All photographs were resized to common dimensions (500 × 357 pixels) and flipped along the horizontal axis to create left-moving and right-moving mirror versions.

Previous research has demonstrated that static images depicting implied motion evoke similar perceptual and neural effects associated with motion processing (Kourtzi and Kanwisher, 2000; Kable et al., 2002; Winawer et al., 2008; Gorman et al., 2011, 2012). For example, prior research using blood-oxygen-level-dependent (BOLD) functional magnetic resonance imaging (fMRI) showed the same patterns of activation in motion regions of the brain when viewing static images depicting motion versus videos of motion (Kourtzi and Kanwisher, 2000; Kable et al., 2002; Winawer et al., 2008). In addition, Gorman et al. (2012) showed similar patterns of performance between officials making judgments on static versus video stimuli. A norming study (*n* = 5) with non-athlete participants verified the directionality implicit in each photo and ensured that information about directionality was evident from stimuli exposed for 500 ms. Stimuli were presented on a laptop computer. Participants pressed the left arrow key if the picture depicted leftward motion and the right arrow key if it depicted rightward motion. The best 95 pairs (190 total pictures including the original and mirrored versions) out of a total of 124 stimuli obtained rating above 85% agreement for directionality. These stimuli were selected for use in the present experiment. The present study used approximately 33% fewer stimuli than the prior soccer study (Kranjec et al., 2010). To obtain comparable power, we increased our sample size to 16, as compared to 12 in the previous study.

### Procedure

Procedures were identical to the prior soccer study (Kranjec et al., 2010). We used a go/no-go (i.e., respond or do not respond) task to mimic the type of decision made in a real game. Participants were informed that they would view a number of confrontations between offensive and defensive players on a laptop computer. They were required to press the spacebar if the defending player committed pass interference according to NCAA/NFL standards. In contrast, they were told to not respond if the event did not depict pass interference. To assist in making clear which player was the offensive player, participants were told that the player closest to the ball was always the offensive player. Before practice trials, all participants were given a bullet-point version of the NFL/NCAA rules for pass interference to review (NFL Digest of Rules, 2013). There was no time limit for review of the rules. There were 20 practice trials and 190 experimental trials. Trials consisted of a 2000 ms fixation cross, followed by a football image presented for 500 ms, and finally a 3000 ms response screen. Participants were presented with all 95 pictures in both directions (leftward versus rightward). Stimuli were presented in two equal blocks of 95 trials. To minimize the possibility of participants noticing the direction manipulation, only one version of each stimulus pair (i.e., rightward or leftward) was randomly presented in the first block of the experiment. The opposing version was presented in random order in the second block of the experiment. Participants responded with one hand (e.g., left or right) for the first half of trials, and the other hand for the last half, to avoid handedness biases. Order of hand use was counterbalanced across participants.

### Analyses

To verify the manipulation of expertise in the present study, the influence of expertise on decision-making was analyzed using a 3-way ANOVA with the number of pass interference calls (Foul Calls) as the dependent variable and Expertise Group (Group) as the independent variable. Alpha level was set at 0.05. To analyze the influence of expertise on spatial biases, data were analyzed using a 3 × 2 ANOVA with Pass Interference Calls as the dependent variable and Expertise Group (Group) and Direction of Motion (Direction) as independent variables. Planned comparisons were performed using paired and independent *t*-tests.

## Results

### Football Knowledge and Experience

#### Football Naïve Group

Performance on the eight multiple-choice questions confirmed that participants possessed little to no knowledge of the official rules of the game (e.g., “At what yard line is the ball snapped for an extra point kick in the NFL?”: average % correct = 6.25%), and facts pertaining to professional teams and players (e.g., “Which of the following teams does Reggie Bush play for?”: average % correct = 18.75%). Overall accuracy on the multiple-choice questions was 29.4 ± 21%. Football Naïve participants had no formal experience playing American football on an organized team (e.g., high school) or any experience officiating American football games at any level of play. Participants reported little to no playing or watching experience on four agreement rating questions using a 5-point Likert scale (e.g., “I have played a lot of organized football games”: mean agreement = 1; “I have watched many football games on television”: mean agreement = 1.5).

#### Football Player Group

Performance on the eight multiple-choice questions confirmed that participants possessed a basic knowledge of the official rules of the game (e.g., “At what yard line is the ball snapped for an extra point kick in the NFL?”: average % correct = 100%) and knew facts pertaining to professional teams and players (e.g., “Which of the following teams does Reggie Bush play for?”: average % correct = 100%). Overall accuracy on the multiple-choice questions was 86%. Football Player participants had no experience officiating American football at any level of play. Further, they demonstrated considerable playing and watching experience on four agreement rating questions using a 5-point Likert scale (e.g., “I have played a lot of organized football games”: mean agreement = 5; “I have watched many football games on television”: mean agreement = 4.9).

#### Football Officials Group

Performance on the eight multiple-choice questions confirmed that the Football Official group possessed a basic knowledge of the official rules of the game (e.g., “At what yard line is the ball snapped for an extra point kick in the NFL?”: average % correct = 100%) and knew facts pertaining to professional teams and players (e.g., “Which of the following teams does Reggie Bush play for?”: average % correct = 100%). Overall accuracy on the multiple-choice questions was 90%. Officials demonstrated that they had considerable watching experience, and moderate playing experience based on four agreement rating questions using a 5-point Likert scale (e.g., “I have played a lot of organized football games”: mean agreement = 3.7; “I have watched many football games on television”: mean agreement = 5.0). Performance on the additional short answer officiating questions demonstrated that participants in the Football Official group had an average of 19.25 years of officiating experience at the college level (SD = 9.5).

### Sports Decision-Making

#### Football Expertise

**FIGURE 1:**
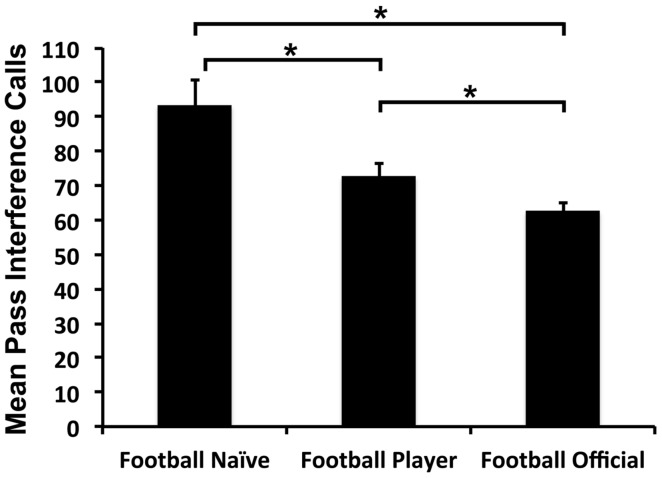
**Average number of pass interference calls for each group.** ^∗^*p* < 0.05.

The three-way ANOVA demonstrated a significant effect of Expertise Group on the total number of pass interference calls made by participants [*F*_(2,48)_ = 10.4, *p* < 0.001, $\eta_{p}^{2}$ = 0.316, **Figure 1**]. Independent *t*-tests demonstrated that the Football Official group made significantly fewer pass interference calls (62.5 calls) than either the Football Naïve (93.3 calls; *t* = 4.0, DF = 30, *p* < 0.001, Cohen’s *d* = 1.4; Cohen’s *d* of 0.2 = small effect size, 0.5 = moderate, 0.8, or greater = large) or Football Player (72.6 calls; *t* = 2.3, DF = 30, *p* = 0.02, Cohen’s *d* = 0.84) groups. In contrast, while the Football Players Group made significantly more pass interference calls than the Football Officials, they made significantly fewer pass interference calls than the Football Naïve Group (*t* = 2.5, DF = 30, *p* = 0.01, Cohen’s *d* = 0.91).

#### Spatial Bias

**FIGURE 2:**
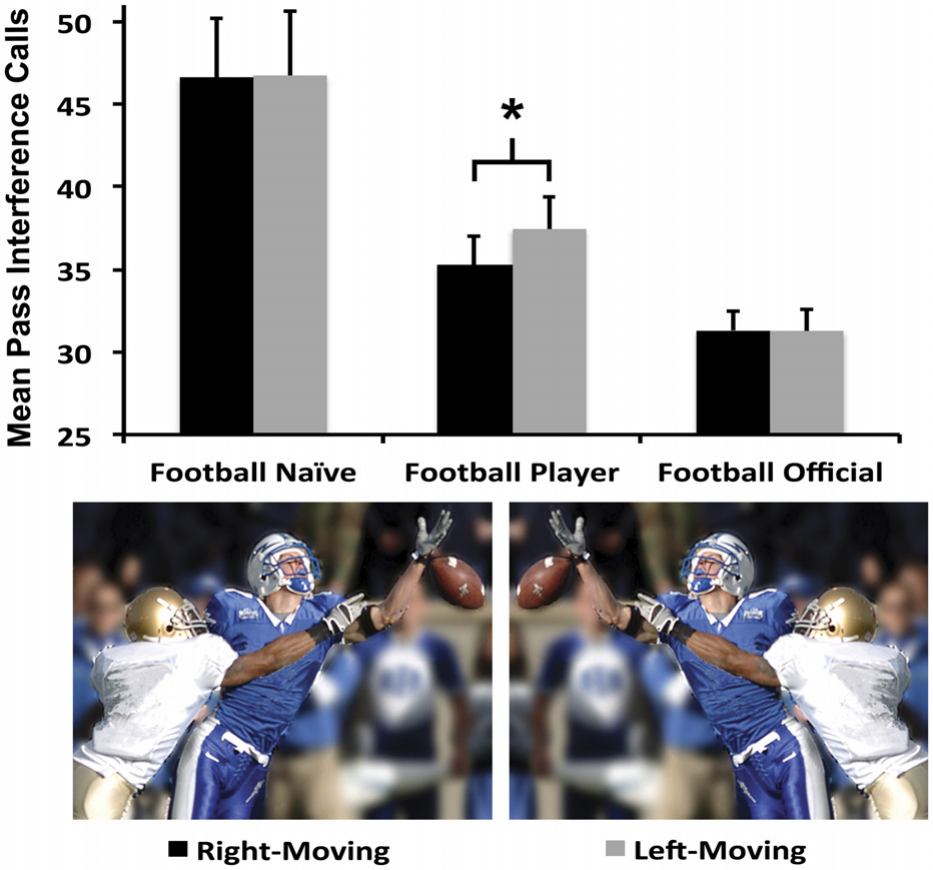
**Average number of right-moving versus left-moving pass interference calls for each group with two example stimuli.** ^∗^*p* < 0.05.

While the 3 × 2 ANOVA demonstrated a significant effect of Group [*F*_(2,96)_ = 19.4, *p* < 0.001, $\eta_{p}^{2}$ = 0.30, **Figure 2**], there was neither a significant main effect of Direction nor a Group × Direction interaction (*F*’s < 0.13.1, *p* > 0.05). Paired samples *t*-tests of the number of pass interference calls made on right-moving versus left-moving plays demonstrated that only the Football Player Group was significantly influenced by spatial biases related to the direction of motion in events (*t* = -2.4, DF = 15, *p* = 0.03, Cohen’s *d* = 0.27), judging more left-moving stimuli to contain pass interference (37.4 calls) than the same stimuli moving rightward (35.2 calls). Thus, the Football Player Group was approximately 6% more likely to call pass interference when seeing a picture in its leftward compared to rightward version, even though the two stimuli were otherwise perceptually identical. Neither the Football Naïve nor Football Official Groups were influenced by spatial biases from the direction of motion (*t*’s < 0.035, DF’s = 15, *p*’s > 0.94; Mean rightward/leftward calls: Naïve = 46.62/46.68, Officials = 31.9/31.8).

## Discussion

The present study hypothesized that decision-making criterion for pass interference in American football would change as a function of expertise (Hypothesis 1). Increased levels of experience with American football was associated with progressively fewer penalty calls, with Football Naïve participants making the most pass interference calls, and Football Officials the least. Football Naïve participants had little to no experience with concepts central to pass interference in American football. Although provided with the official criteria used for pass interference judgments in the NFL, they had limited experience applying these concepts in interpreting events. Thus, participants had little to no domain-specific decision-making expertise to apply to the possible pass interference events. In contrast, both the Football Player and Football Official groups have years of experience with American football and knowledge of the pass interference rule. While both groups have substantial experience and knowledge of pass interference, officials undergo 100s of hours of training to make split-second decisions in ambiguous situations (Referee Enterprises, 2012). Furthermore, they have a significant number of hours applying these decisions on the field of play. Specifically, officials possess domain-specific expertise that can be applied to the task in the current study. While players are intimately knowledgeable with the concept of pass interference, they lack specific experience making pass interference calls on the field of play. Unlike players, officials are trained to evaluate pass interference by applying a stringent set of categories, or “philosophies,” to each call on the field (e.g., pass interference: arm bar, obstruction of path, etc.; Referee Enterprises, 2012). One of these specified categories must be met for a penalty call to be made on the field. Furthermore, for each call, the official making the penalty call must report to the head referee exactly which category was met and how it was met (Referee Enterprises, 2012). Thus, officials are trained to decompose ambiguous events into clear categories to disambiguate information occurring during a brief interval of time, rather than simply evaluating an overall impression of the event. These factors represent an important component of the officials’ domain-specific decision-making expertise and provide clear criteria for decision-making. This specific training represents a critical difference between our groups, and may play a significant role in the findings from the current study. Future studies obtaining data from players and officials with matched levels of experience as players, but differing only by experience with training as an official will provide better insight into the importance of this training in the present findings.

As the level of play increases from high school to the NCAA to the NFL, the numbers of categories officials are trained to use also increases (Referee Enterprises, 2012; NFL Digest of Rules, 2013). Thus, experience at one level of play does not necessarily imply expertise at another level of play. However, research supporting near-transfer of expertise in decision-making (Rosalie and Müller, 2014; Loffing et al., 2015) suggests that prior experience officiating at other levels of play may transfer.

Nonetheless, inexperienced officials may still suffer from increased susceptibility to increased rates of penalty calling (Holmes, 2012; Seifert, 2012; Staff, 2012; Wade, 2012). Officiating crews are typically comprised of an experienced core of officials, with only one “rookie” official on the field (Holmes, 2012; NFL Digest of Rules, 2013). Thus, the potential impact of a single official inexperienced at a given level of play is minimized by the current system. Unfortunately, entire officiating crews during the 2012 NFL season were often comprised of “rookie” replacement officials with no experience officiating in the NFL (Holmes, 2012)– providing a potential foundation for errors in officiating decisions.

In addition, the current study found that college-level players of American football were more likely to call pass interference when pictures of player confrontations depicted leftward compared to rightward motion (Hypothesis 2). However, neither Football Naïve participants nor expert football officials demonstrated a significant spatial bias when making pass interference calls (Hypothesis 3). Directional biases are evident in how we conceptualize events broadly (Chatterjee, 2011), and generalize to aspects of language (Chatterjee, 2001), memory (Halpern and Kelly, 1993), esthetics (Christman, 1995; Maass et al., 2007) and social attributes (Chatterjee, 2002; Suitner and Maass, 2007) that we attribute to participants of events. Prior research posits that the development of spatial biases may be a product of culturally relative visual habits like reading direction (Tversky et al., 1991; Chatterjee, 2001; Maass and Russo, 2003; Dobel et al., 2007; Woods et al., 2013), body-specific motor habits related to handedness (Casasanto, 2011) or the brain’s lateralization of spatial and attentional processing (Chatterjee et al., 1995). These data may also suggest a failure to transfer decision-making skills learned as a player to those required as an official. Future studies investigating the potential influence of handedness and other possible causes of spatial bias in the context of decision-making and expertise will further our understanding of mechanism(s) underlying the bias found in the current study.

As fans and players, we may disagree with officiating decisions on the field. While sometimes we may be correct, the same experience that gives us a love for the game may also makes us susceptible to spatial biases on the field of play. Our data suggest that spatial biases are modulated by the degree of experience participants have applying knowledge of pass interference to sports decisions in ambiguous events; however, at least some degree of experience with American football is necessary to have a prototypical representation of these events. We suspect that naïve participants do not have such a representation and are thus not susceptible to a directional perceptual bias, whereas expert referees are able to mitigate their perceptual bias by applying formal categorization rules in making officiating decisions.

One potential limitation in the current study relates of the use of static images depicting motion, rather than videos of motion events. While prior research demonstrates the validity of this method (Kourtzi and Kanwisher, 2000; Kable et al., 2002; Winawer et al., 2008; Gorman et al., 2012), officials are faced with viewing live events that may be more difficult to process than a static image presented for a brief period of time. In addition, future research investigating the absolute accuracy of penalty calls, using stimuli specifically chosen based on the presence or absence of each specific pass interference criteria, as well as combinations of these criteria would provide further insight into the interaction between expertise and decision-making in officiating.

In summary, we found that expertise with American football has two effects on the decision to make a pass interference call. First, greater experience is associated with fewer overall interference calls. Second, experience is associated with an inverted *U*-shaped susceptibility to a directional bias in making these interference calls. This directional bias may play an important role in how knowledgeable non-expert viewers perceive any sport with frequent contact between opposing players (basketball, hockey, etc.) in which events are ambiguous.

Findings from the current study have important implications for decision-making within a variety of sports and in everyday life. For example, as a person drives a car, he, or she is faced with rapidly integrating perceptual and cognitive information to successfully make numerous decisions with real-world consequences. This is true in many situations. While we may consider ourselves experts in a domain (e.g., driving, penalty calls, etc.), the absence of rigorous domain-specific training may make us more susceptible to biases in our decisions. At the very least, our data demonstrate that the level of a person’s expertise in a domain significantly impacts decision-making criteria and may play an important role in the interpretation of events on, and perhaps off, the field of play.

## Author Contributions

Conceived and designed the experiments: AW, AK, ML, AC. Performed the experiments: AW, ML. Analyzed the data: AW. Wrote the paper: AW, AK, AC.

## Conflict of Interest Statement

The authors declare that the research was conducted in the absence of any commercial or financial relationships that could be construed as a potential conflict of interest.

## Footnotes

1 For a glossary of American football terminology please see: http://www.nfl-360.com/glossary.
