# Supplementary material for: Expertise and decision-making in American football
Source: Front Psychol. 2015 Jul 13;6:994. doi: 10.3389/fpsyg.2015.00994 (PMC4499674; doi:10.3389/fpsyg.2015.00994)
Supplement: Supplementary file 1 [file Image_1.PDF]

Which of the following is considered to be an offsides penalty?

a) an offensive lineman moves prior to the snap
b) a defensive player crosses the line of scrimmage and contacts an offensive player before the ball is snapped
c) a defensive lineman moves prior to the snap
d) an offensive lineman crosses the line of scrimmage and contacts a defensive player before the ball is snapped

What happens if a player downs the ball, willingly or unwillingly, behind their own goal line?
a) touchback
b) touchdown
c) first-down
d) safety

On what yard line is an extra point kicked from?
a) 15 yards
b) 20 yards
c) 25 yards
d) 30 yards

In an official game of football how many players play per side?

a) 9
b) 10
c) 11
d) 12

Reggie Bush currently plays for which professional team?

a) Miami Dolphins
b) Baltimore Ravens
c) New Orleans Saints
d) San Francisco 49'ers

Which city will host the 2012 Superbowl?

a) Chicago
b) Los Angeles
c) Indianapolis
d) Newark

The Cardinals are a professional football team in what state?

a) Missouri
b) Arizona
c) New York
d) Kansas

Which of the following colors is a home color for the Cincinnati Bengals?

a) orange
b) blue
c) yellow
d) red

Please rate your agreement with the following statement on a scale of 1 through 5
(5= strongly agree).

I have watched many football games on television.

Please rate your agreement with the following statement on a scale of 1 through 5
(5= strongly agree).

I have watched many football games in a stadium.

Please rate your agreement with the following statement on a scale of 1 through 5
(5= strongly agree).

I have played a lot of informal football games (weekend pick up games, back yard games, etc.).

Please rate your agreement with the following statement on a scale of 1 through 5
(5= strongly agree).

I have played a lot of organized football games (high school, college, etc.).
